# COVID-19 Health Impact: A Use Case for Syndromic Surveillance System Monitoring Based on Primary Care Patient Registries in the Netherlands

**DOI:** 10.2196/53368

**Published:** 2024-09-26

**Authors:** Imme Rahmon, Mark Bosmans, Christos Baliatsas, Mariette Hooiveld, Elske Marra, Michel Dückers

**Affiliations:** 1 Faculty of Behavioral and Social Sciences, University of Groningen, Grote Kruisstraat 2/1, Groningen, 9712 TS, the Netherlands, 31 (0)50-363 6419; 2 Department of Health, Living Environment, and Aftercare, National Institute for Public Health and the Environment, Bilthoven, the Netherlands; 3 Department of Disasters and Environmental Hazards, Nivel, Utrecht, the Netherlands; 4 ARQ Centre of Expertise for the Impact of Disasters and Crises, ARQ National Psychotrauma Center, Diemen, the Netherlands

**Keywords:** SARS-CoV-2, epidemic surveillance, public health, general practice, disaster health research

## Abstract

**Background:**

The COVID-19 pandemic challenged societies worldwide. The implementation of mitigation measures to limit the number of SARS-CoV-2 infections resulted in unintended health effects.

**Objective:**

The objective of this study is to demonstrate the use of an existing syndromic surveillance system in primary care during a first series of quarterly cross-sectional monitoring cycles, targeting health problems presented in primary care among Dutch youth since August 2021.

**Methods:**

Aggregated data from the surveillance system of Nivel Primary Care Database were analyzed quarterly to monitor 20 health problems often reported in the aftermath of disasters and environmental incidents. Results were stratified by age (ie, 0‐4, 5‐14, and 15‐24 years), sex, and region (province). Weekly prevalence rates were calculated as the number of persons consulting their general practitioner in a particular week, using the number of enlisted persons as the denominator. Findings were compared to quarterly survey panel data, collected in the context of the Integrated Health Monitor COVID-19, and the Dutch stringency index values, indicative of the intensity of COVID-19 mitigation measures.

**Results:**

Over time, weekly rates pointed to an increased number of consultations for depressive feelings and suicide (attempts) among youth, during and after periods with intensified domestic restrictions.

**Conclusions:**

The results illustrate how, from a disaster health research perspective based on the COVID-19 pandemic, health consequences of pandemics could be successfully followed over time using an existing infrastructure for syndromic surveillance and monitoring. Particular areas of health concern can be defined beforehand, and may be modified or expanded during the monitoring activities to track relevant developments. Although an association between patterns and changes in the strictness of mitigation measures might seem probable, claims about causality should be made with caution.

## Introduction

### Background

As of December 2019, the SARS-CoV-2 virus, known for causing COVID-19, has kept the entire world under its spell. Although most people only experienced mild symptoms, COVID-19 might have contributed to a large number of hospitalizations and mortality among older adults or patients who are chronically ill [1]. In response to the outbreak, the Dutch government imposed various mitigation measures to control its spread, such as hygiene measures and social distancing. Additionally, a total of 3 nationwide lockdowns have been implemented between March 2020 and December 2021 [2]. However, these measures resulted in unintended social, economic, and health effects. Regarding the latter, a frequently observed outcome was an increase in mental health problems among youth [3-5]. A clearer understanding of the probable health impact of the COVID-19 pandemic in the Netherlands would guide decision-making.

This paper aims to demonstrate the feasibility of using an existing syndromic surveillance system to periodically monitor changes in health outcomes among Dutch youth, during and in the aftermath of the COVID-19 pandemic. We describe the methodology and the lessons learned from the first rounds of short-cycle monitoring among the Dutch youth using general practitioner (GP) registries, from August 2021 to September 2022. Monitoring outcomes regarding mental health will serve as an illustration of how GPs’ routine-based registry data can be used for the periodic (quarterly) monitoring of the impact of a public health crisis (ie, the COVID-19 pandemic) on population health.

### The COVID-19 Pandemic in the Netherlands

The Netherlands has a population of 17.6 million inhabitants, of whom 14% were born abroad [6]. Up to April 2022, over 7.9 million SARS-CoV-2 cases have been reported, of which there have been at least 22,000 recorded deaths [7]. However, general population COVID-19 testing for symptomatic individuals only became available in June 2020 [2]. The reported number of SARS-CoV-2 infections is therefore expectedly higher.

The Netherlands experienced its first confirmed SARS-CoV-2 infection on February 27, 2020. The Dutch government initially responded to the COVID-19 pandemic by implementing mitigation measures primarily centered around hygiene practices. Shortly after, a nationwide lockdown was imposed, resulting in the closure of the hospitality industry, schools, and childcare facilities. These restrictions were eased by the end of June, leading to a complete reopening of primary education and public transportation. During this period, the use of face masks became mandatory, and widespread COVID-19 testing facilities were introduced. A resurgence of COVID-19 cases was experienced after the summer, resulting in a second nationwide lockdown during the subsequent holiday season. With the start of the COVID-19 vaccination campaign in January 2021—which initially prioritized health care personnel, older individuals, and high-risk populations—infection rates and hospitalizations decreased significantly, leading to a gradual reopening of society. In December 2021, the emergence of the Omicron variant necessitated the announcement of the third lockdown in the Netherlands. Despite its higher transmissibility, the Omicron variant proved to be less pathogenic than previous variants. Together with the crucial role of the repeat vaccination campaign in herd immunity, the majority of measures had expired by the end of March 2022 [2].

## Methods

### Integrated Health Monitor COVID-19

Data used for this research were obtained through the Integrated Health Monitor COVID-19 [8]. This 5-year health monitor aimed to create a solid knowledge base regarding the physical and mental health effects of the global COVID-19 pandemic on the Dutch population to advise and support local, regional, and national authorities in policy making. This information is considered valuable for the development and deployment of interventions to limit the negative impact of the COVID-19 pandemic on public health. The focal areas of the monitor are being refined and adjusted based on expert consultations and findings from annual literature reviews, guiding 2 types of monitoring: short- and long-cycle monitoring. Both types of monitoring are based on survey-collected data and electronic health record data from GPs. Long-cycle monitoring gives the most in-depth overview of the health status of the population and particular vulnerable groups. Survey and GP registration data are combined with other data sources on, for instance, virus infections, social demography (ie, income, migration background), and particular risk factors (ie, chronic mental or physical health problems, social support, lifestyle), enabling subgroup comparisons. Since long-cycle monitoring depends on retrospectively created datasets and its analysis is time-intensive, it is at odds with the objective of rapid sense making. Short-cycle monitoring, instead, allows for generating policy information with a shorter interval, yet it lacks the possibility to enrich survey or registry data with other data sources [9-12]. During the health monitor, GP registry data were obtained from an existing syndromic surveillance system, embedded within the Nivel Primary Care Database (Nivel-PCD) infrastructure. Nivel-PCD was previously used to monitor the health impact of disasters and major crises in the Netherlands [9-12].

Internationally, syndromic surveillance has been successfully used in analyzing the health impact of terrorist attacks [13,14] and armed conflicts [15], as well as natural disasters [16-18]. Furthermore, it has also proven valuable in epidemiology, enabling the identification of the beginning of the autumn/winter wave of the influenza pandemic [19]. Concerning the aftermath of the COVID-19 pandemic, several publications mention the use of syndromic surveillance systems to retrospectively explore changes in primary health care consultations [20-26]. This retrospective use of syndromic surveillance data hinders the ability to effectively communicate with local, regional, and national policy makers and professionals during times of pandemic or other significant crises, thereby impeding the use of syndromic surveillance for immediate response and resource allocation.

### Nivel Primary Care Database (Nivel-PCD)

Data were obtained from an existing syndromic surveillance system, embedded within the Nivel-PCD, used to monitor the number of weekly GP consultations for the early detection of (un)expected changes in public health. Diagnoses during the consultations were registered by GPs in electronic health records using the first edition of the International Classification of Primary Care (ICPC) [27]. The ICPC allows symptoms and nondisease conditions from the patient’s perspective (eg, general weakness/tiredness) in primary care to be included [28]. Data were collected from about 390 participating general practices, with approximately 1.6 million registered persons. This accounted for 9% of the Dutch population and comprised a nationally representative sample.

### Short-Cycle Monitoring

As part of the short-cycle monitoring, data on GP consultations were obtained to secure policy formation with a shorter interval. The first cycle of the monitor was performed using data from August to October 2021 (youth only), the second from November to December 2021 (youth only), the third from January to March 2022 (all age groups), the fourth from April to June 2022 (all age groups), and the fifth from July to September 2022 (all age groups). Monitoring will be continued until early 2026. Every 3 months, the outcomes are presented on public websites, serving the purpose of informing public health policy making in the short term [29]. This paper will only discuss or provide detailed explanations about the findings related to youth health.

The monitoring was based on an a priori selection of 20 health problems typically experienced in the aftermath of a disaster or as a reaction to a potential environmental threat [30], of which the weekly number of patients with consultations for these health problems was collected and reported on quarterly. These symptoms can often be nonspecific in nature (regarding etiology) and have a psychosomatic component. Under the current circumstances, symptoms such as headaches, sleeping problems, and fatigue can be approached separately or as part of a long-lasting or chronic condition, or other cause [30]. A subset of these complaints is recognized as symptoms of the post–COVID-19 condition [31]. Because of troubling signals from other (international) research [32-34], the Dutch suicide hotline [35], and official suicide figures regarding unusually high levels of suicide (attempts) and suicidal thoughts among the youth [36], we also included GP consultations for suicide (attempts) in addition to the 20 common health symptoms. The analyzed symptoms and complaints and their ICPC codes are displayed in Multimedia Appendix 1.

### Ethical Considerations

The use of electronic health records for research purposes is allowed under certain conditions. When these conditions are fulfilled, approval by a medical ethics committee is not obligatory for this type of observational study containing no directly identifiable data (art. 24 GDPR implementation Act jo art. 9.2 sub j GDPR). This study was approved according to the governance code of Nivel-PCD (NZR-00321.036, NZR-00322.009 and NZR-00323.018).

### Analysis

The weekly prevalence for each of the selected symptoms was calculated as the number of persons for whom the relevant diagnosis was registered in medical records during a GP consultation in a given week, divided by the total number of registered persons in the general practices, expressed per 100,000. Prevalence rates were stratified by age groups (ie, 0‐4, 5‐14, and 15‐24 years), sex, and region (province). The results were plotted as a time series together with an earlier (reference) prepandemic year for visual inspection, using the ggplot2 (version 3.5.0) package in RStudio (version 4.3.2, R Core Team) [37,38].

## Results

The most frequently occurring mental health problems between August 2021 and September 2022 are described below to illustrate the usability of periodic (quarterly) monitoring of routinely registered GP consultations to study the impact of the COVID-19 pandemic on population health. Although the subset of health problems studied also comprised physical complaints, only mental health problems shown to have had the biggest impact on youth mental health during the COVID-19 pandemic will be discussed in the following paragraphs.

### Psychological Complaints

**Figure 1.**
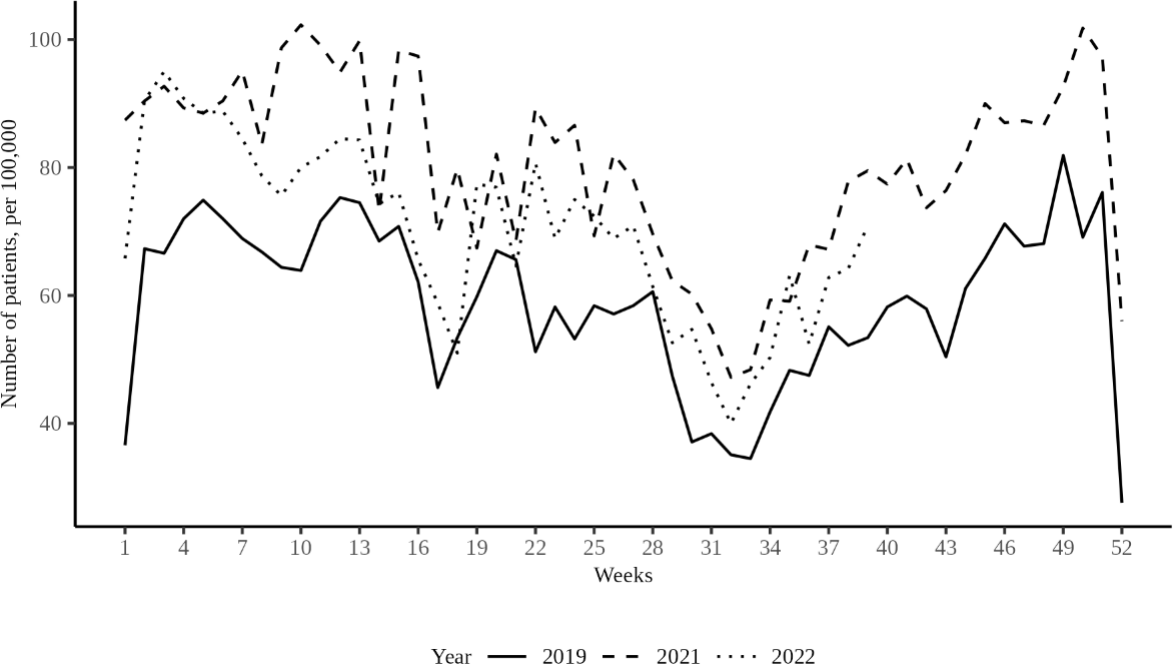
Weekly number of patients (aged 0‐24 years) who visited their general practitioner for depressive feelings in the Netherlands between January and December 2021 and 2022, compared to 2019 as a baseline reference.

In the period of August to October 2021, an increase was observed in the prevalence of mental health problems—especially feelings of depression—compared to the same period of previous years [39]. Over time, the largest peak in the demand for health care for depressive feelings was observed during the third lockdown in mid-December 2021. Due to a notable rise in depressive feelings during the same period in previous years, the disparity between 2021 and prior years appeared comparatively less pronounced than it had been earlier in 2021 [40]. Depressive feelings almost exclusively concern those aged 15‐24 years and females [41-43]. Figure 1 shows the weekly number of patients (aged 0‐24 years) that visited their GP for depressive feelings in the Netherlands between January and December 2021 and 2022, compared to 2019 as a baseline reference.

Simultaneously, increases in the prevalence of other psychological complaints (ie, anxiety and severe stress) were seen in August to October 2021 when compared to previous years [39]. From November to December 2021, small increases were observed for these complaints among 15‐24-year-olds [40]. Nevertheless, the increases observed for these complaints were less profound compared to those seen for depressive feelings, and the number of GP consultations for these issues displayed greater variability. The weekly number of patients (aged 0‐24 years) who visited their GP for anxiety or severe stress in the Netherlands between January and December 2021 and 2022, compared to 2019 as a baseline reference, is displayed in Multimedia Appendices 2 and 3.

### Suicide (Attempts)

**Figure 2.**
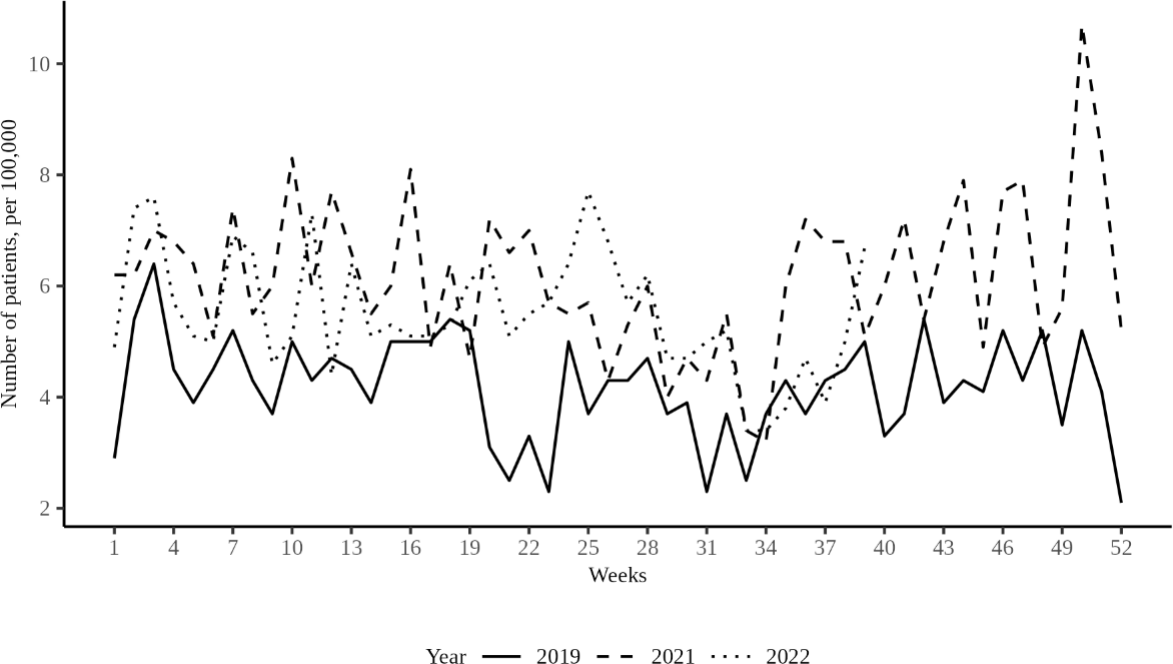
Weekly number of patients (aged 0‐24 years) who visited their general practitioner for suicide (attempts) in the Netherlands between January and December 2021 and 2022, compared to 2019 as a baseline reference.

Although the number of persons with these complaints is relatively low, the average weekly prevalence of GP consultations for suicide (attempts)—which include suicidal thoughts and attempts with and without fatal outcomes—had increased by over 36% on average in 2021 compared to 2019. This trend remained stable at the beginning of 2022: from January to March, the number of consultations regarding suicide (attempts) was 37% higher compared to the same period in 2019. The largest peak was observed from April to June 2022, during which the number of GP consultations was 39% higher [41]. These high rates were exclusively seen among the group aged 15‐24 years [42]. However, there was a decline observed during the third quarter of 2022 (from July to September), with a noticeable 18% increase in consultations for suicide (attempts) compared to the corresponding period in 2019. It is important to consider that these figures might be influenced by the summer holidays, as consultations for suicide (attempts) tend to be less frequent during this time of year [43]. Figure 2 shows the weekly number of patients (aged 0‐24 years) that visited their GP for suicide (attempts) in the Netherlands between January and December 2021 and 2022, compared to 2019 as a baseline reference.

### Risk Groups

Those aged 15‐24 years visited the GP more often for mental or neurological complaints (eg, anxiety, depression, dizziness, fatigue, and memory or concentration problems). Results also showed a generally higher prevalence of the discussed symptoms in females. This was especially the case for psychological problems (eg, anxiety, depression, and severe stress), but also for stress-related presenting complaints (ie, fatigue, headaches, dizziness, palpitations, and nausea) [39-43].

## Discussion

### Principal Findings

This study shows how an existing syndrome surveillance system, based on routinely registered GP consultations incorporated into a broader monitoring program, has enabled monitoring of the potential health impact of the COVID-19 pandemic on Dutch youth. By focusing on several relevant health outcomes using GP registries, the development of particular areas of health concern linked to the virus and the mitigation measures could be monitored over time.

Examples derived from these figures include the heightened prevalence of depressive feelings and suicide (attempts) among Dutch youth. Several international systematic literature reviews on the health consequences of COVID-19 support these findings, as they report an increase in the prevalence of mental health symptoms (ie, depressive feelings, anxiety, stress, and suicide and self-harm behaviors) in youth during and after the pandemic [44-51]. Concerning the impact of COVID-19 lockdown on child and adolescent mental health, a significant increase in depressive feelings, anxiety, and stress was observed among children during lockdown—especially among those with previous or current psychological health complaints—compared to rates observed before lockdown [44]. Other reviews stress the possible worsening of the COVID-19 lockdown on youth mental health, expressed in a rise in mental health concerns and suicidal behaviors [45,48]. Comparable patterns emerged from the short-cycle survey data collected from approximately 5000 individuals (aged 12‐25 years) per quarterly round since September 2021. The data consistently showed a negative long-term impact of the COVID-19 pandemic on psychological well-being and suicidal ideation. Furthermore, the data revealed a succession of lockdown periods by a steep increase in these mental health indicators [29]. Although only part of the self-reported complaints are translated into actual GP consultations, these observations show that changes in people’s self-reported health and well-being regarding the COVID-19 pandemic are reflected in the number of persons visiting their GP. The use of GP registration data is therefore a faster and more representative tool to monitor population health, compared to the use of data obtained through (quarterly) surveys.

Both developments—the increased prevalence of depressive feelings and suicide (attempts) among Dutch youth—are logically attributable to COVID-19 measures. In relation to the Dutch stringency index—a measure based on 9 of the response metrics (ie, school/workplace and public transportation closures, stay-at-home requirements, and restrictions regarding public events), indicating the intensity of mitigation measures implemented at a certain time point during the COVID-19 pandemic [52]—the observed prevalence of depressive feelings and suicide (attempts) from GP registries was particularly elevated succeeding the third lockdown in December 2021 (see Multimedia Appendix 4). Yet causality needs to be approached with caution, as we could not correct for alternative mechanisms and confounders. However, where the surveillance system allows weekly generation of health data for syndromic surveillance from a subset of participating GPs from Nivel-PCD, which is crucial for short-cycle monitoring, long-cycle monitoring involves all Nivel-PCD GPs [53]. This facilitates comparisons across various vulnerable groups at different times for more comprehensive analyses. Despite this, comparable outcomes were observed between these 2 monitoring approaches.

Nevertheless, although not completely attributable to the COVID-19 pandemic, these results highlight the urgent need for fitting policies to lower the negative mental health impact on youth in today’s society. As we will likely face comparable threats in the future that will affect mental health, it is important to invest in a sustainable and resilient society.

### Implications for Research and Society

During the COVID-19 pandemic, governments worldwide were challenged to find a balance between implementing public health measures to mitigate the spread of the disease and simultaneously mitigating the adverse public health impact of the mitigation measures on public health. In the interest of mapping the development of population health and well-being during and after the COVID-19 crisis—a timeline with multiple potential health risks, ranging from virus infections to public mitigation measures—reliable and timely monitoring approaches are indispensable to public health authorities and practitioners. Monitoring data can guide decision-making, under the realization that it is notoriously complex to attribute (developments in) health status to external factors, whether pandemic-related or not.

Throughout the last 2 decades, several population-based monitoring systems have been implemented by public health institutions in the Netherlands. Large-scale questionnaires are currently used to get insight into the health (perception) of Dutch citizens and to offer public health authorities the opportunity to respond to signals of negative health effects. The addition of quarterly reports on GP consultations to these existing public health monitors during times of crisis provides a better understanding of midterm changes in public health on a much higher frequency. This knowledge proved to serve as an important basis for timely public health policy making under the (post–)COVID-19 circumstances and is expected to do so for future public health emergencies.

Under the condition that the GP functions as a bridge between primary and secondary care, this way of monitoring is therefore suitable in case of any future pandemic or disaster. Defining areas of health concern beforehand based on the type of disaster—whether natural (including naturally occurring epidemics) or human-made—and subsequently conducting a longitudinal monitoring of these measures will provide an understanding of its consequences for public health.

To the best of our knowledge, articles discussing the use of existing syndromic surveillance systems for periodic monitoring and documenting of health outcomes during the COVID-19 pandemic are lacking. In disaster-health research, emergency department (ED) syndromic surveillance systems have become widely employed, as they provide crucial insights into population health during the acute phase of a disaster, aiding in the immediate response and resource allocation. Also, throughout the unfolding COVID-19 pandemic, ED surveillance has been demonstrated to be of significant value in examining its impact on public health [54,55]. However, its effectiveness diminishes when it comes to monitoring the aftermath of such a creeping crisis. Moreover, while many studies have retrospectively examined the impact of the COVID-19 pandemic on population health [20-26], this retrospective approach is primarily effective for understanding previous pandemics or forecasting short- and long-term consequences of coming potential pandemics. It falls short of regularly informing policy makers and professionals on changes in the prevalence of health outcomes during times of crisis, which hinders the timely generation of fitting policies and effective interventions in response to emerging trends in public health.

### Limitations

This monitoring approach demonstrates several methodological strengths that contribute to its reliability and applicability. One of the key strengths lies in the ease of use and adaptability of the monitoring. The use of this well-established primary care data infrastructure eliminates any burden on persons or GPs. Additionally, this monitoring is based on an a priori selection of health symptoms commonly experienced in the aftermath of disasters or in response to potential environmental threats. A subset of these symptoms is recognized as the long-term effects of COVID-19. Regardless of the type of disaster, this approach can include the surveillance of additional relevant outcomes, such as suicide attempts in this context, complementing the assessment of health symptoms.

Concerning the reliability and extensiveness of the data, various notable strengths emerge. The data collected is based on the professional clinical judgment and diagnoses made by GPs, ensuring a more trustworthy and unbiased source of information compared to other data sources (eg, self-reported complaints through surveys). Moreover, the data exhibits a high level of quality and completeness, further enhancing its reliability and usefulness for comprehensive analysis and informed decision-making. Another notable strength is the longitudinal availability of the data, spanning from 2011 onwards. This extensive dataset enables robust comparisons and trend analysis, providing valuable insights into changes and patterns over time.

The generalizability of the results to the Dutch population is another important strength. The registry data from over 390 participating GPs in the Netherlands covers approximately 1.6 million citizens, accounting for 9% of the Dutch population. This data serves as an ideal resource for monitoring changes in population health, as new health problems are initially presented at this primary care level. Additionally, the use of the universally adopted ICPC classification system by all Dutch GPs ensures the generation of representative data on primary care throughout the Netherlands.

In terms of reporting frequency, the weekly prevalence of symptoms and associated patterns is compared to previous years as a baseline reference. Findings are reported on a 3-month basis, aligning with the timeline of the quarterly survey panels. This reporting frequency was considered sufficient but can be further increased if necessary. By providing quarterly reports, this monitoring system ensures interim insights into the current public health situation in the Netherlands, allowing for a faster policy response to emerging trends compared to the traditional annual monitoring cycle, which focuses on extensive analyses of vulnerable groups using combined data sources. Additionally, repeated data collection enables the tracking of health and support needs trends over time.

Lastly, the use of a broader monitoring program enables the comparison and supplementation of findings with other data pillars within the program, including quantitative survey data collected through various research methods (ie, variation in recruitment method, study sample, questionnaires, and measurement times) and qualitative data gathered from open-ended questions in surveys, interviews, and focus groups. This approach can yield in-depth information and offer explanations for the observed changes in health outcomes as a consequence of the COVID-19 pandemic. Although currently unavailable within the Integrated Health Monitor COVID-19, data from other sources on the same topic during the COVID-19 pandemic (eg, the number of internet searches for words like “depression” or “suicide,” or the number of specialist diagnoses) would also be useful to include in future short-cycle monitoring.

Nonetheless, short-cycle monitoring of GP registries has some limitations. The data collected for this monitoring are recorded for patient care, and therefore observational. Whether or not health problems are registered as a symptom or diagnosis can differ between GPs. Moreover, no distinction was made between medically unexplained symptoms—symptoms that occur in the absence of disease or that are expressed disproportionately for the underlying illness—and symptoms that do meet the existing diagnostic criteria for diseases or disorders. Therefore, it would be useful to involve GPs in commenting on results obtained in future short-cycle monitoring through, for instance, focus groups or surveys.

Although we successfully tracked health care utilization for both physical and mental health complaints across all stages of the pandemic, certain phases experienced changes in health care seeking behavior, which may have led to potential underreporting within GP registries. For instance, during the first months of the pandemic, the closure of GP practices due to illness or quarantine of personnel or health care avoidance by patients due to the advice of the government to limit GP visits unless urgent has led to a decline in health care utilization as of March 2020. In previous Nivel reports, it has been demonstrated that health care utilization for both acute and chronic conditions experienced a decline during the first year of the COVID-19 pandemic [56,57]. Additionally, there was a decrease in the number of prescriptions issued by GPs compared to 2019 [58]. During this period, the prevalence of complaints among Dutch youth is less certain. From 2021 onwards, health care use had returned to prepandemic levels and higher (as seen for depressive feelings and suicide (attempts). Although the number of GP visits may have been underestimated due to the implementation of lockdown measures, it is anticipated that care avoidance would be less frequent in more severe cases. Within the context of the Integrated Health Monitor COVID-19, the potential limitation of underestimation is mitigated through the incorporation of (quarterly) surveys, in which health issues are assessed via self-reporting.

Another important limitation is the inability to combine the data with other data sources that are needed to identify vulnerable groups (eg, socioeconomic status or migration background). Although collected by the Dutch Central Bureau of Statistics, these data are not yet available for short-cycle monitoring. However, long-cycle monitoring of GP registries does provide an opportunity to compare different vulnerable groups at different moments in time, which allows for more in-depth analyses [53], whereas short-cycle monitoring provides insights into more general trends at the population level.

Lastly, it is noteworthy to state that we could not designate any of the complaints studied due to the SARS-CoV-2 infection. Although a subset of the nonspecific symptoms was flagged as possible post–COVID-19 symptoms, no direct link could be made as the occurrence of infection was not registered, and these symptoms were mostly also common in the population before the pandemic.

### Conclusions

From a disaster health research perspective, the health consequences of the COVID-19 pandemic could be successfully followed over time using an existing infrastructure for syndromic surveillance and monitoring. To ensure the meaningfulness of such monitoring, a publicly accessible stepped care health care system with national coverage is required. In cases where the presence of GPs acting as gatekeepers to the secondary health care system is lacking, data obtained from hospitals’ ED syndromic surveillance systems can be used as an alternative to provide a representative picture of population health. The pilot described here served as a meaningful contribution to the monitoring toolbox during the COVID-19 pandemic. Therefore, this approach holds significant potential as a foundation for future disaster and environmental health research, by guiding public health policy making and managing potential public health emergencies.

## Supplementary material

10.2196/53368 Multimedia Appendix 1 List of nonspecific symptoms and matching ICPC-codes based on the SaP questionnaire.

10.2196/53368 Multimedia Appendix 2 Weekly number of patients (aged 0-24 years) that visited their general practitioner for anxiety in the Netherlands between January and December 2021 and 2022, compared to 2019 as a baseline reference.

10.2196/53368 Multimedia Appendix 3 Weekly number of patients (aged 0-24 years) that visited their general practitioner for stress in the Netherlands between January and December 2021 and 2022, compared to 2019 as a baseline reference.

10.2196/53368 Multimedia Appendix 4 The Dutch stringency index values indicative for the intensity of COVID-19 mitigation measures, from March 2020 to January 2023.

## Abbreviations

ED: emergency department
GP: general practitioner
ICPC: International Classification of Primary Care
Nivel-PCD: Nivel Primary Care Database
