# Supplementary material for: COVID-19 Health Impact: A Use Case for Syndromic Surveillance System Monitoring Based on Primary Care Patient Registries in the Netherlands
Source: JMIR Public Health Surveill. 2024 Sep 26;10:e53368. doi: 10.2196/53368 (PMC11611799; doi:10.2196/53368)
Supplement: Multimedia Appendix 1 [file publichealth-v10-e53368-s001.pdf]

Table 1. List of non-specific symptoms and matching ICPC-codes based on the SaP questionnaire[30].

Symptoms	ICPC-code (name code)
Shortness of breath or shortness of breath at rest (without exertion)[a]	R02 (Dyspnea/shortness of breath attributed to the airways)<br>R03 (Wheezing)<br>R04 (Other breathing problems)<br>R29 (Other respiratory symptoms/ complaints)
Fatigue[a]	A04 (Fatigue/weakness)
Muscle pain[a]	L18
Nausea	D09
Chest pain or pressure[a]	K01 (Pain attributed to heart)<br>K02 (Pressure/tightness attributed to heart)<br>K03 (Other pain attributed to cardiovascular system)
Palpitations[a]	K04 (Patches/heartbeat awareness)
Feeling anxious/nervous/tense	P01
Sudden (severe) stress or crisis	P02 (Crisis/transient stress response)
Depressive feelings[a]	P03 (Feeling down/depressed)
Sleeping problems	P06 (Insomnia/other sleep disorder)
Headache[a]	N01<br>N02 (Tension headache)
Dizziness or lightheadedness	N17
Memory or concentration problems[a]	P20 (Memory/concentration/orientation disorders)
Other abnormality(s) of smell/taste[a]	N16

[a]*Symptoms relevant for post-COVID-19 condition according to the WHO[31].*
