# Supplementary figures and images for: COVID-19 Health Impact: A Use Case for Syndromic Surveillance System Monitoring Based on Primary Care Patient Registries in the Netherlands
Source: JMIR Public Health Surveill. 2024 Sep 26;10:e53368. doi: 10.2196/53368 (PMC11611799; doi:10.2196/53368)

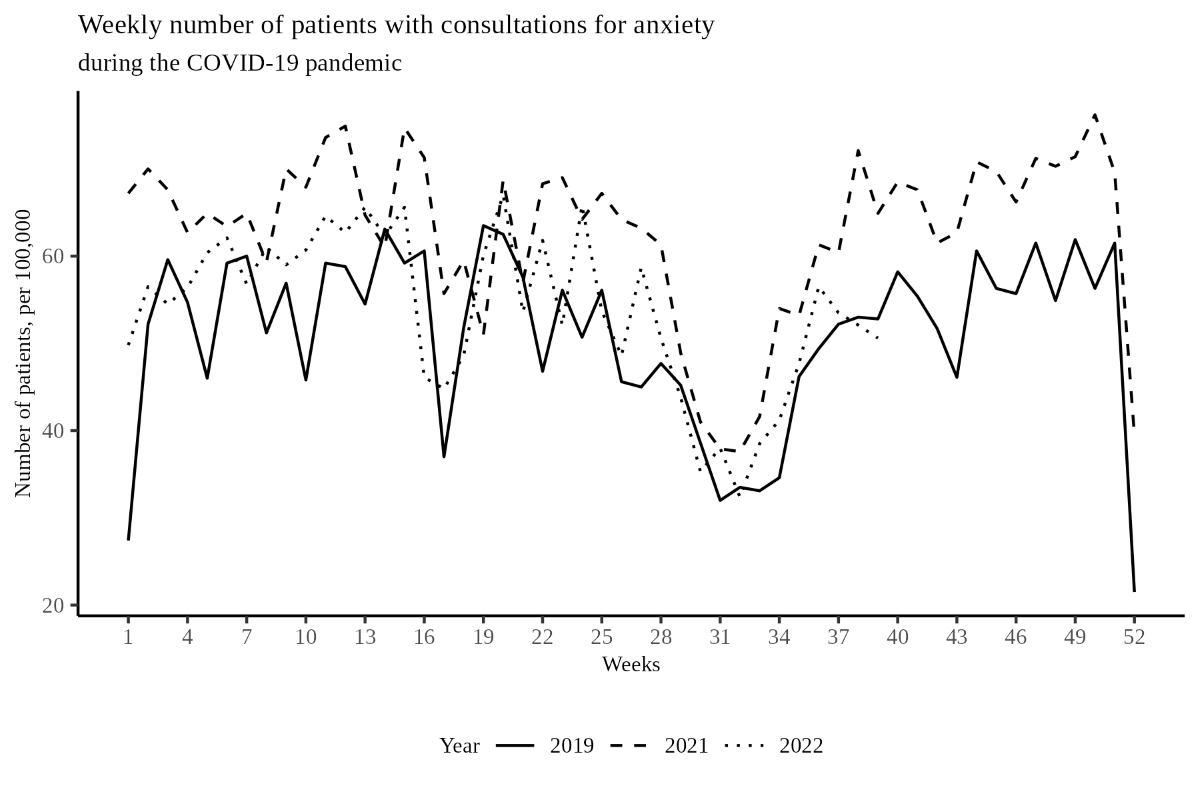

Supplement: Multimedia Appendix 2 [file publichealth-v10-e53368-s002.png]

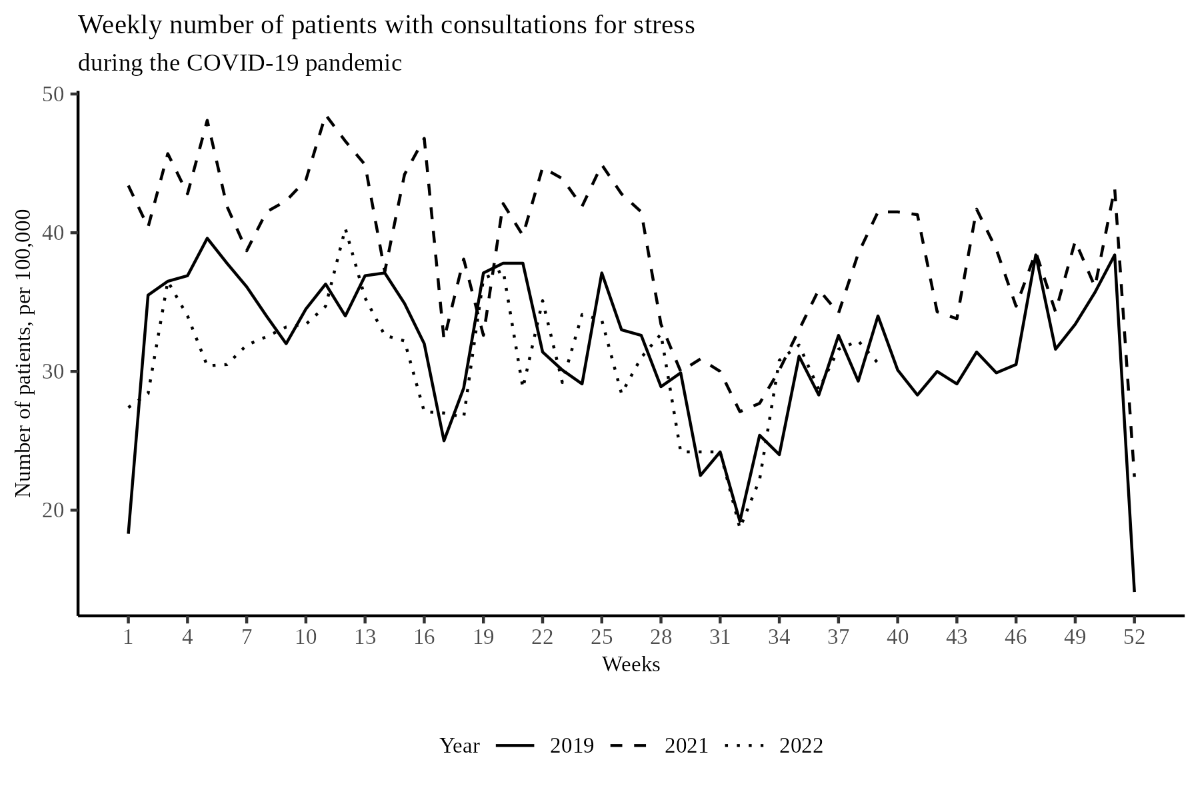

Supplement: Multimedia Appendix 3 [file publichealth-v10-e53368-s003.png]

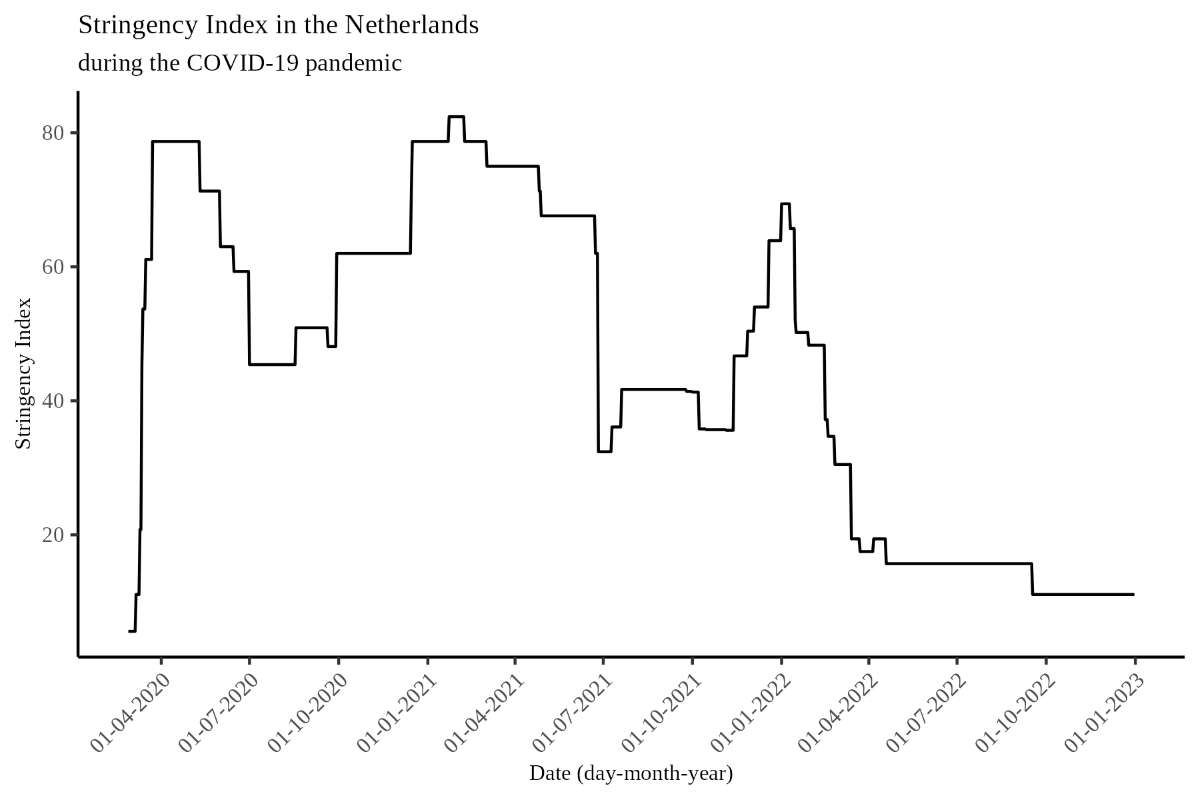

Supplement: Multimedia Appendix 4 [file publichealth-v10-e53368-s004.png]
